# Implementation and costs of housing adaptations among older adults with different functional limitations in Japan

**DOI:** 10.1186/s12877-022-03100-9

**Published:** 2022-05-20

**Authors:** Rumiko Tsuchiya-Ito, Shota Hamada, Björn Slaug, Ayako Ninomiya, Kazuaki Uda, Tomoaki Ishibashi

**Affiliations:** 1 Research Department, Institute for Health Economics and Policy, Association for Health Economics Research and Social Insurance and Welfare, Tokyo, Japan; 2 Dia Foundation for Research On Ageing Societies, Tokyo, Japan; 3 Department of Health Services Research, Faculty of Medicine, University of Tsukuba, Ibaraki, Japan; 4 Department of Home Care Medicine, Graduate School of Medicine, The University of Tokyo, Tokyo, Japan; 5 Department of Health Sciences, Faculty of Medicine, Lund University, Lund, Sweden

**Keywords:** Accessibility, Claims data, Housing adaptations, Costs, Disability

## Abstract

**Background:**

Accessible housing is crucial to maintain a good quality of life for older adults with functional limitations, and housing adaptations are instrumental in resolving accessibility problems. It is unclear to what extent older adults, who have a high risk of further functional decline, use housing adaptation grants acquired through the long-term care (LTC) insurance systems. This study aimed to examine the utilization of housing adaptation grants in terms of implementation and costs, for older adults with different types of functional limitations related to accessibility problems.

**Methods:**

The study sample included individuals from a suburban city in the Tokyo metropolitan area who were certified for care support levels (indicative of the need for preventive care) for the first time between 2010 and 2018 (N = 10,372). We followed the study participants over 12 months since the care needs certification. We matched and utilized three datasets containing the same individual’s data: 1) care needs certification for LTC insurance, 2) insurance premium levels, and 3) LTC insurance claims. We conducted a multivariable logistic regression analysis to estimate the likelihood of individuals with different functional limitations of having housing adaptations implemented. Afterward, we conducted a subgroup analysis of only older adults implementing housing adaptation grants to compare costs between groups with different functional limitations using the Mann–Whitney U and Kruskal–Wallis tests.

**Results:**

Housing adaptations were implemented among 15.6% (*n* = 1,622) of the study sample, and the median cost per individual was 1,287 USD. Individuals with lower extremity impairment or poor balance were more likely to implement housing adaptations (adjusted odds ratio (AOR) = 1.290 to AOR = 2.176), while those with visual impairment or lower cognitive function were less likely to implement housing adaptations (AOR = 0.553 to AOR = 0.861). Costs were significantly lower for individuals with visual impairment (1,180 USD) compared to others (1,300 USD).

**Conclusion:**

Older adults with visual or cognitive limitations may not receive appropriate housing adaptations, despite their high risk of accessibility problems. Housing adaptation grants should include various types of services that meet the needs of older people with different disabilities, and the results indicate there may be a need to improve the system.

**Supplementary Information:**

The online version contains supplementary material available at 10.1186/s12877-022-03100-9.

## Background

“Aging in place” has been defined as “individuals growing old in their own homes, with an emphasis on using an environmental modification to compensate for limitations and disabilities” [1–3]. It is commonly considered desirable to promote aging in place, and more than 65% of middle-aged and older adults in Japan prefer to live at home even if they need long-term care (LTC) support [3].

For older adults with disabilities, it is important that the housing environment is designed or adapted to compensate for their disabilities to maximize their ability to attain aging in place [4]. Previous research has demonstrated the significance of housing adaptations for community-dwelling older adults with disabilities, with regard to different health aspects. For instance, although some reviews suggest that the evidence is not consistent [5, 6], several studies report that housing adaptations prevent injuries from falls [7, 8]. Housing adaptations have also been associated with maintenance or improvement of level of meeting LTC needs [10, 11], reduction of risk of low quality of life [11], and lowered risk of mortality [12].

Furthermore, it is important that housing adaptations be focused and carefully planned for older adults with disabilities, who have difficulties in performing daily activities because of accessibility problems. Accessibility is an aspect of person–environment fit and denotes the relationship between an individual’s functional capacity (competence) and the demands or design of the physical environment [4, 13]. It entails that even if the individual’s functional competence deteriorates, the capacity for activity can be improved by lowering the demands of the environment, by implementing changes such as housing adaptations [14]. As most of the current housing stock in Japan is not built to fit the needs of people with functional limitations [15], there is a high demand for such housing adaptations. The Statistics Bureau of Japan recently reported that only 42.4% of households with older adults reach what is defined as a “standard level” of barrier-free design, which is handrails at more than two places or no level difference indoors concretely. Moreover, only 8.8% of households reach a “high level” of barrier-free design, that is, handrails at more than two places, no level difference indoors, and corridors wide enough to pass with a wheelchair [16]. Several functional conditions that produce accessibility problems have been reported, such as physical and visual impairment [14]. Illustrating the actual use of housing adaptations will provide insight into the challenges met by older adults with functional limitations, when using them to improve accessibility.

In Japan, a housing adaptation grant using LTC insurance, which is public insurance for people who need preventive care or LTC services, is the typical way for older adults to adapt their housing environment to match their physical functions. Older adults who are certified for care support levels do not require LTC services immediately, but this group is considered at risk of functional deterioration in the near future and is recommended to use preventive care [17]. They are therefore considered an important target group for housing adaptations that could serve to maximize their abilities, counter functional deterioration, and thereby maintain a good quality of life.

This housing adaptation grant supports older adults financially with up to 200,000 JPY (1,883 USD; 1USD = 106.19 JPY on March 31, 2018), and older adults only have to pay 10% or 20% of the housing adaptation costs [18]. These housing adaptation grants can be used any number of times as long as the cumulative amount does not exceed the maximum limit of 200,000 JPY. Housing adaptation grants are limited to cover five types of adaptations designed to meet the needs of older adults with difficulty in basic functions of mobility [19]: 1) installation of handrails or grab bars, 2) elimination of level differences, 3) change of floor materials, 4) change of doors, and 5) change of lavatory basins; and additionally, other adaptive or reconstructive measures that accompany any of these five types of housing adaptations. These LTC services are available in the same way throughout Japan. The expenditures of LTC insurance are supported by the social security system [20], and it is important to ensure that the resources available for housing adaptations should be distributed to those in need of them. However, there is limited evidence on these issues, which are of particular importance for Japan as a super-aging society [21]; therefore, we need to examine the current situation of the housing adaptations from the perspective of different functional limitations related to accessibility problems. The aim of the present study was thus to examine the utilization of housing adaptation grants in terms of implementation and costs, for older adults with different types of functional limitations related to accessibility problems. Clarifying the costs will help us understand the effectiveness and shortcomings of housing adaptation grants for older adults with functional limitations.

## Methods

### Study design and setting

This study had a retrospective cohort design and utilized data from residents of a Japanese suburban city in the Tokyo metropolitan area. The population of this city was about 490,000 (data from 2018), and older adults aged ≥ 65 years accounted for 27.4%, which was comparable with the national average of 28.1% [22].

### Data sources

We used three separate datasets with data from April 2007–March 2018, including 1) care needs certification for LTC insurance, 2) insurance premium levels, and 3) LTC insurance claims. These datasets are stored in each municipality across Japan to keep a record of the Japanese LTC insurance system. Each residents' data is stored in each dataset according to the type of information as follows.

The three datasets comprise:

- 1) Data on care needs certification for LTC insurance, including each individual’s certified levels and assessment results from 74 items on the severity of physical disabilities and cognitive impairments used to assess LTC needs [23, 24]. Each beneficiary’s certified level is determined by applying a computer-based algorithm to the test results of physical disabilities and cognitive impairments. Additionally, a review by a panel of specialists appointed by the local government is used as well [23]. We used these data to understand beneficiaries’ age, sex, certified levels, specific medical procedures, disability level, and different functional limitations.
- 2) Data on LTC insurance premium levels, which are classified from individual and household-level taxation. The LTC insurance premium levels are used to indicate household income, which we have used as a proxy for economic status.
- 3) Data on LTC reimbursement claims including information on the cost of housing adaptation grants and assistive devices. We utilized data on the implementation and costs of housing adaptation grants based on reimbursement claims.

We were granted access to these datasets in accordance with an agreement from a collaborative research project between this municipality and the Dia Foundation for Research on Ageing Societies, Tokyo. All data were linked using the pre-assigned anonymous identifying numbers.

### Study sample

The study sample comprised residents aged ≥ 65 years old who had been certified for care support levels in LTC insurance for the first time by the local government between April 2010 and March 2018. In Japanese LTC insurance system, the eligibility criteria are for 65 years or older, or those who are 40–64 years of age with any of 16 specific diseases. These people can apply for the certification, and are classified into one of seven levels (care support level 1 or 2, care need levels 1–5); we focused only on older adults with care support level 1 or 2. We regarded certification as being the first for an individual if we found no existing certification data between April 2007 and March 2010, because care needs certification is generally reviewed every two years. From the initial sample (*n* = 11,229), we excluded those younger than 65 years old (*n* = 46), those lacking data on LTC insurance premium levels *(n* = 62), and those who were excluded from certification because they moved out of the municipality within 12 months of the certification or because of their death (*n* = 749). With these exclusions, 10,372 individuals were included in the final study sample.

### Conceptual framework and variables

**Fig. 1:**
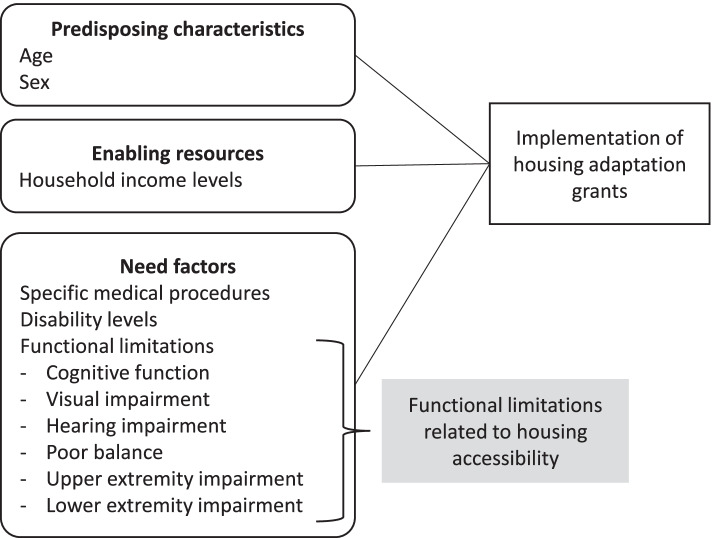
Conceptual framework of this study

To select variables for the analysis of housing adaption grant use, we applied a conceptual framework based on Andersen’s behavioral model [25–27] and on previous studies [28–34], (see Fig. 1). Andersen’s frequently used behavioral model is useful to comprehensively elucidate the factors related to the process of health service utilization, including the utilization of LTC services [27]. It includes *predisposing characteristics*, *enabling resources,* and *need factors* as factors related to health service use [25]. Enabling resources are the conditions which permit an individual or family to act on a value to satisfy a need regarding health service utilization [25]. Need factors refer to the individual’s self-perceived illness or the severity of illness as clinically judged by professionals [25].

### Predisposing characteristics

Age and sex were selected as predisposing factors based on the review of previous research [30–32]. Age was grouped into three categories: 65–74 years old, 75–84 years old, and ≥ 85 years old [35]. These variables were derived from care needs certification for LTC insurance data.

### Enabling resources

Household income was selected as an enabling resource based on the review of previous research [28–30, 33, 34], because those with higher socioeconomic status could pay for services and access more information on them, and also socioeconomic status was related to greater use of health or long-term care services in previous studies [29, 30]. Household income levels were determined by each beneficiary’s LTC insurance premium level, which ranged from persons receiving public assistance (i.e., living below the poverty line as determined by the national government and receiving financial support for all medical and LTC service expenditures under public assistance programs) [36], level 1 (all members of the household have a total annual income ≤ 800,000 JPY [7,533 USD]) to level 18 (persons who have an individual total annual income ≥ 20,000,000 JPY [188,341 USD]). Level 5 (persons who are not taxed individually but have family members within the same household paying taxes) is the standard household income level where standard premium rates apply. Household income was categorized into four categories: receiving public assistance, low (levels 1–4), middle (level 5), and high household income (levels 6–18). This variable was taken from LTC insurance premium levels data.

### Need factors

Based on the review of previous research, we selected specific medical procedures, disability levels, and functional limitations as need factors. Andersen [25] mentions two types of need factors: evaluated needs and perceived needs; we focused on evaluated needs in this study. Evaluated needs are objectively assessed, and are important because they may represent the most immediate cause of health service use [25]. All variables comprising need factors were taken from care needs certification for LTC insurance data.

Data regarding specific medical procedures during the last 14 days were collected in the assessment of care needs certification. The 12 types of medical procedures provided mainly by nurses included intravenous cannulation, total parenteral nutrition, dialysis, stoma, oxygen therapy, mechanical ventilation, treatment of bronchotomy, treatment for pain, tubal feeding, monitoring (e.g., blood pressure, heart rate, and oxygen saturation), treatment of pressure ulcers, and catheterization. Medical procedures were categorized as “received” if at least one of these types of medical procedures had been provided. Specific medical procedures were used because medical backgrounds might be important to decide the implementation of housing adaptation grants to recognize effects on their disabilities and prognosis, as with other LTC services [29, 31]. However, we did not have access to data on medical backgrounds, such as medical claims, and we only use medical procedures as a variable related to the medical background in the data on care needs certification.

Disability levels were also selected, referring to the previous research [29, 30, 32] and assessed using the “degree of independent daily living for older people with disabilities”; individuals were categorized as “independent” (independent or Level J1: some disabilities, but daily living is mostly independent, capable of going outdoors with means of transportation, etc.), “Level J2” (some disabilities, but daily living is mostly independent, capable of going out near home), “Level A1” (indoor living and predominantly independent, goes out with assistance, spends most time during the daytime out of bed), “Level A2” (Indoor living and predominantly independent, but unable to go out without assistance and does not go out frequently; repeating cycles of lying down and getting up from bed during the daytime), or “Level B1 or worse” (Level B: some assistance needed for indoor living, also lies in bed for much of the daytime, although sitting position is possible; Level C: bedridden all day, requires assistance with excretion/urination, meals, and dressing/undressing) [37].

Functional limitations related to housing accessibility are referred to in the previous reports [14, 38]. The detailed categories of functional limitations were cognitive function, visual impairment, hearing impairment, poor balance, upper extremity impairment, and lower extremity impairment. We used data on care needs certification for LTC insurance and applied these categories. The application process is shown in Supplementary Information 1. The application of these categories and confirmation of content validity was checked by a researcher with more than ten years of clinical experience as a physical therapist and a researcher who is the author of previous reports of functional limitations [14].

Cognitive function was assessed based on the nationally standardized methods designated by the Japanese Ministry of Health, Labour and Welfare, and was measured as the “degree of independent daily living for older adults with dementia” [37]. Cognitive function was categorized into “independent,” “Rank I” (the patient has some type of dementia, but is almost independent in terms of daily living at home and in society), or “Rank IIa or worse” (for example, Rank IIa: some daily life-disturbing symptoms, behaviors, and problems in communication are seen, but the individual can lead daily life independently if kept watch on by someone when outside the home; Rank M: marked psychiatric symptoms/related symptoms or serious physical disorders requiring expert management).

Visual impairment was ascertained from four ranked answers and categorized into “intact” or “visually impaired” (“can see figure to check visual acuity from 1 m distance” to “hardly see anything”). Hearing impairment was also ascertained from four ranked answers and categorized into “intact” or “hearing impaired” (“can hear as much as the regular volume of sound” to “hardly hear anything”). Poor balance was assessed as to whether the individuals can keep standing and divided into three categories: “can keep standing without some support,” “can keep standing with some support,” or “cannot keep standing.” Upper extremity impairment was assessed dichotomously based on whether the patient had paralysis in the upper extremities on the right or left side. Upper extremity impairment was categorized as “upper extremity impaired” if at least one item was applicable. Lower extremity impairment was assessed in the same way and was categorized as “lower extremity impaired” if at least one item was applicable.

### Housing adaptation data

Implementation of housing adaptation was categorized into implemented or not implemented based on the existence of reimbursement claims data. We selected the study sample at the date of care needs certification, and we decided to implement or not implement housing adaptations during the 12-month follow-up. We defined the index year as the one containing the month with the date of care needs certification. The reason we focused on the 12 months after certification was that the maximum valid duration of the first certification was 12 months [39]. The costs were also used for the data on reimbursement claims data, and JPY was converted to USD using the exchange rate on March 31, 2018 (1 USD = 106.19 JPY) [40]. The cost included only the municipality’s payments, not individuals’ out-of-pocket payments.

### Statistical analysis

To examine the associations between functional limitations related to housing accessibility with the implementation of the housing adaptation grants, we determined the number and percentage of individuals for each characteristic. These functional limitations were compared to implement or not implemented housing adaptations using the χ^2^ test.

Subsequently, we used a multivariable logistic regression analysis to estimate the likelihood of implementation of housing adaptations, illustrated by the odds ratio (OR) with a 95% confidence interval (95% CI). The dependent variables were the implementation of housing adaptation grants; independent variables were functional limitations related to housing accessibility. As covariates, we included predisposing characteristics (age and sex), enabling resources (household income level), and need factors (specific medical procedures and disability levels). In addition, there was concern that the results would change over time during these nine years, although the basic system of housing adaptation grants has not changed. We conducted a sensitivity analysis to check the difference in the first and last three years (2010–2012 and 2015–2018).

Later, we conducted a subgroup analysis and extracted only the sample who implemented housing adaptation grants to examine whether the costs were different by their functional limitations. The costs were compared using the Mann–Whitney U test and Kruskal–Wallis test. Multiple comparisons with the statistically significant independent variables were conducted using Dunn’s test with a Bonferroni adjustment [41]. SPSS version 25.0 (SPSS Inc., Chicago, IL) was used for all analyses, and the significance threshold was set at *p* = 0.05 (two-tailed).

## Results

Of the total study sample, 15.6% (*n* = 1,622) had housing adaptations implemented in the year after the care needs certification. The housing adaptation usage the second time comprised 6.2% of individuals in this study’s data. The distribution of implementation by month is shown in Supplementary Information 2. On average, the first housing adaptation occurred 4.0 ± 2.7 months (range: 1–12 months) after certification. The second month after certification had the highest relative frequency (26.8%), and the distribution was right-skewed. Most individuals used the categories of 175,000 to 187,500JPY, which includes the maximum amount of costs (180,000 JPY) paid per housing adaptation in Supplementary Information 3.

A comparison of predisposing, enabling, and need factors, except for functional limitations, between individuals who implemented housing adaptations and those who did not is provided in Table 1. Males, individuals receiving public assistance, at care support level 1, and required medical procedures were less likely to implement housing adaptations, while those with lower disability levels were more likely to implement housing adaptations.

**Table 1:** Implementation of housing adaptations, by year, certified care support level, and other individual factors

	Total n (%) *n* = 10,372	Implemented n (%) *n* = 1622	Not implemented n (%) *n* = 8750	*p*-value
Index year				
Year 2010	1209 (11.7)	205 (12.6)	1004 (11.5)	0.191
Year 2011	972 (9.4)	138 (8.5)	834 (9.5)	
Year 2012	1149 (11.1)	161 (9.9)	988 (11.3)	
Year 2013	1246 (12.0)	185 (11.4)	1061 (12.1)	
Year 2014	1323 (12.8)	213 (13.1)	1110 (12.7)	
Year 2015	1289 (12.4)	216 (13.3)	1073 (12.3)	
Year 2016	1258 (12.1)	214 (13.2)	1044 (11.9)	
Year 2017	1384 (13.3)	217 (13.4)	1167 (13.3)	
Year 2018	542 (5.2)	73 (4.5)	469 (5.4)	
Certified level				
Care support level 1	5892 (56.8)	824 (50.8)	5068 (57.9)	** < 0.001**
Care support level 2	4480 (43.2)	798 (49.2)	3682 (42.1)	
**Predisposing characteristics**				
Age				
65 to 74 years old	2464 (23.8)	388 (23.9)	2076 (23.7)	0.097
75 to 84 years old	5689 (54.8)	919 (56.7)	4770 (54.5)	
≥ 85 years old	2219 (21.4)	315 (19.4)	1904 (21.8)	
Sex				
Male	3880 (37.4)	551 (34.0)	3329 (38.0)	**0.002**
Female	6492 (62.6)	1071 (66.0)	5421 (62.0)	
**Enabling resources**				
Household income level				
Person receiving public assistance	689 (6.6)	21 (1.3)	668 (7.6)	** < 0.001**
Low (Level 1 to 4)	5470 (52.7)	880 (54.3)	4590 (52.5)	
Middle (Level 5; standard)	956 (9.2)	177 (10.9)	779 (8.9)	
High (Level 6 to 18)	3257 (31.4)	544 (33.5)	2713 (31.0)	
**Need factors**				
Specific medical procedures in the last 14 days at LTC needs assessment				
Not received	9866 (95.1)	1570 (96.8)	8296 (94.8)	**0.001**
Received	506 (4.9)	52 (3.2)	454 (5.2)	
Disability levels				
Independent to level J1	577 (5.6)	72 (4.4)	505 (5.8)	** < 0.001**
Level J2	4845 (46.7)	679 (41.9)	4166 (47.6)	
Level A1	2378 (22.9)	431 (26.6)	1947 (22.3)	
Level A2	2317 (22.3)	370 (22.8)	1947 (22.3)	
Level B1 or worse	255 (2.5)	70 (4.3)	185 (2.1)	

χ^2^ test. Significant *p*-values are bolded

The association of functional limitations with the implementation of housing adaptations is shown in Table 2. Those with lower cognitive function and visually impaired were less likely to implement housing adaptations. Those with poor balance or lower extremity impairment, on the other hand, were significantly more likely to implement housing adaptations.

**Table 2:** Implementation of housing adaptations, by functional limitations

Functional limitation	Total n (%)	Implemented n (%)	Not Implemented n (%)	*p*-value
Cognitive function				
Independent	4797 (46.2)	874 (53.9)	3923 (44.8)	** < 0.001**
Rank I	4688 (45.2)	664 (40.9)	4024 (46.0)	
Rank IIa or worse	887 (8.6)	84 (5.2)	803 (9.2)	
Visual impairment				
Intact	8514 (82.1)	1373 (84.6)	7141 (81.6)	**0.003**
Visually impaired	1858 (17.9)	249 (15.4)	1609 (18.4)	
Hearing impairment				
Intact	6731 (64.9)	1083 (66.8)	5648 (64.5)	0.085
Hearing impaired	3641 (35.1)	539 (33.2)	3102 (35.5)	
Poor balance				
Can keep standing without any support	1619 (15.6)	147 (9.1)	1472 (16.8)	** < 0.001**
Can keep standing with some support	8297 (80.0)	1367 (84.3)	6930 (79.2)	
Cannot keep standing	456 (4.4)	108 (6.7)	348 (4.0)	
Upper extremity impairment				
Intact	9489 (91.5)	1474 (90.9)	8015 (91.6)	0.337
Upper extremity impaired	883 (8.5)	148 (9.1)	735 (8.4)	
Lower extremity impairment				
Intact	6893 (66.5)	951 (58.6)	5942 (67.9)	** < 0.001**
Lower extremity impaired	3479 (33.5)	671 (41.4)	2808 (32.1)	

χ^2^ test. Significant *p*-values are bolded

The association of functional limitations related to housing accessibility with the implementation of housing adaptation after adjusting for covariates is shown in Table 3. Regarding functional limitations, the multivariable logistic regression analysis showed that individuals with lower cognitive function in Rank I (adjusted OR: 0.774; 95% CI: 0.690–0.868) and Rank IIa or worse (adjusted OR: 0.553; 95% CI: 0.434–0.704), and those who were visually impaired (adjusted OR: 0.861; 95% CI: 0.741–0.999) were less likely to implement housing adaptations. However, individuals whose balance was categorized as “can keep standing with some support” (adjusted OR: 1.724; 95% CI: 1.429–2.080) and “cannot keep standing” (adjusted OR: 2.176; 95% CI: 1.608–2.945), and those with lower extremity impairment (adjusted OR: 1.290; 95% CI: 1.148–1.449) were more likely to implement housing adaptations.

**Table 3:** Multivariable logistic regression analysis, estimating the likelihood of having housing adaptations implemented, in relation to functional limitations and other individual factors

	AORs	95% CI		*p*-value
		Lower	Upper	
**Functional limitation**				
Cognitive function (ref: Independent)				
Rank I	**0.774**	0.690	0.868	< 0.001
Rank IIa or worse	**0.553**	0.434	0.704	< 0.001
Visual impairment (ref: Intact)				
Visually impaired	**0.861**	0.741	0.999	0.049
Hearing impairment (ref: Intact)				
Hearing impaired	0.954	0.846	1.076	0.446
Poor balance (ref: Can keep standing without any support)				
Can keep standing with some support	**1.724**	1.429	2.080	< 0.001
Cannot keep standing	**2.176**	1.608	2.945	< 0.001
Upper extremity impairment (ref: Intact)				
Upper extremity impaired	0.972	0.804	1.175	0.768
Lower extremity impairment (ref: Intact)				
Lower extremity impaired	**1.290**	1.148	1.449	< 0.001
**Predisposing characteristic**				
Sex (ref: Male)				
Female	**1.182**	1.026	1.361	0.021
Age (ref: 65 to 74 years old)				
75 to 84 years old	0.986	0.861	1.130	0.838
≥ 85 years old	0.854	0.717	1.016	0.075
**Enabling resource**				
Household income (ref: Middle (Level 5))				
Person receiving public assistance	**0.147**	0.092	0.235	< 0.001
Low (Level 1 to 4)	0.867	0.723	1.039	0.123
High (Level 6 to 18)	1.061	0.866	1.301	0.567
**Need factors**				
Specific medical procedures (ref: Not received)				
Received	**0.644**	0.477	0.869	0.004
Disability levels (ref: Independent to level J1)				
Level J2	1.096	0.841	1.427	0.498
Level A1	**1.341**	1.019	1.764	0.036
Level A2	1.113	0.841	1.473	0.453
Level B1 or worse	**1.947**	1.319	2.872	0.001
c-statistics	0.630	0.616	0.644	

*AOR* adjusted odds ratio, *95% CI* 95% confidence interval. AORs indicating significantly higher (> 1.0) or lower (< 1.0) likelihood for having housing adaptations implemented are bolded

Regarding other factors except for functional limitations, females (adjusted OR: 1.182; 95% CI: 1.026–1.361), and those who had lower disability levels at Level A1 (adjusted OR: 1.341; 95% CI: 1.019–1.764), or Level B1 or worse (adjusted OR: 1.947; 95% CI: 1.319–2.872) were more likely to implement housing adaptations. Individuals receiving public assistance (adjusted OR: 0.147; 95% CI: 0.092–0.235) or receiving specific medical procedures (adjusted OR: 0.644; 95% CI: 0.477–0.869) were less likely to implement housing adaptations.

In a sensitivity analysis comparing the first and last three years (2010–2012 and 2015–2018) with the results of the whole period (2010–2018), the associations of sex, medical procedures, disability levels (only Level A1), visual impairments, and lower limb functions with the use of housing adaptations did not reach the threshold for statistical significance. However, the estimates were similar across all periods.

The differences in housing adaptation costs among individuals who implemented housing adaptations are shown in Table 4. The median housing adaptation cost was 1,287 USD. Regarding functional limitations, individuals with visual impairment (median cost: 1,180 USD) had significantly lower costs from housing adaptations compared to other individuals (median cost: 1,300 USD). Regarding other factors except for functional limitations, individuals aged 85 years or older (median cost: 1,245 USD) compared to those 65–74 years old (median cost: 1,384 USD), and those receiving public assistance (median cost: 684 USD) compared to other household income categories (median cost of low: 1,292 USD, middle: 1,203 USD, high: 1,335 USD), had significantly lower costs from housing adaptations.

**Table 4:**
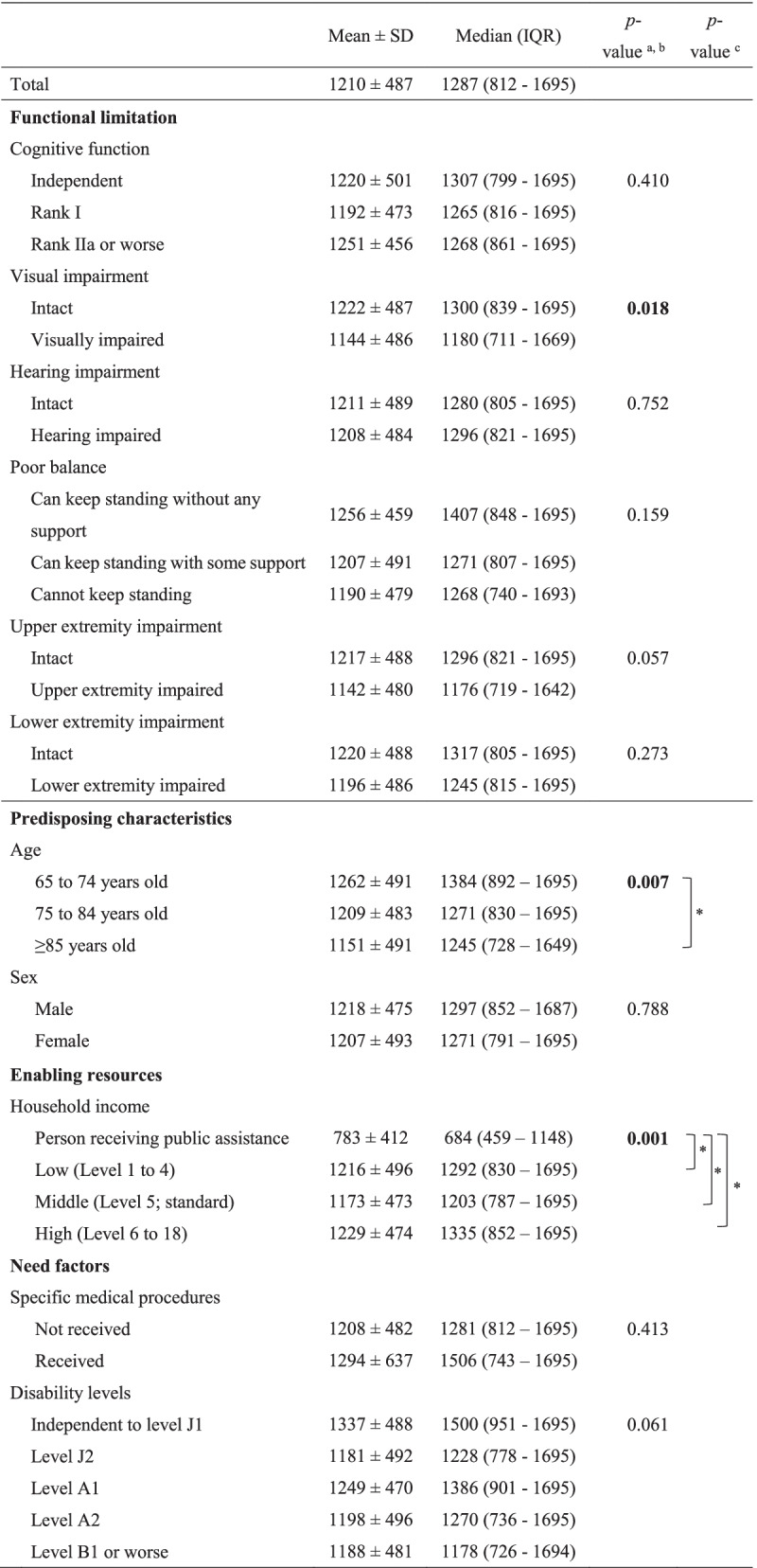
Comparison of costs for implemented housing adaptations (*n* = 1622), by functional limitations and other individual factors

^a^Mann–Whitney U test; ^b^Kruskal–Wallis test; ^c^Multiple comparison by Dunn’s test with Bonferroni adjustment was conducted as a post hoc test, and only statistically significant results are reported (*p < 0.05). Significant *p*-values are bolded

## Discussion

This study was undertaken to address whether the current utilization of housing adaptations among older adults in Japan differed depending on the types of functional limitations. To the best of our knowledge, this is the first study to examine the associations of various functional limitations with the implementation and costs of housing adaptations in Japan.

At first, poor balance and lower extremity impairment substantially increased the likelihood of having housing adaptations implemented. Poor balance and lower extremity impairment affect mobility significantly [42]. As the five types of housing adaptations covered by the grant are specifically designed to meet the needs of older adults with difficulty in basic functions of mobility [19], the results indicate that the intent of the national Ministry of Health, Labour and Welfare in this respect has been achieved. As a national social security system, housing adaptation grants in LTC insurance are appropriately used for older adults with disability of mobility.

Notably, older adults with lower cognitive function or visual impairment were less likely to implement housing adaptations, and especially older adults with visual impairment paid significantly less for housing adaptations. There are few studies on the effect of housing accessibility problems on people with cognitive impairments [43]. With regard to housing adaptations for people with visual impairments, however, some research suggests they may slightly reduce the risk of falls after one year [44]. These two types of functional limitations are not components of walking ability per se, but they affect how the individual recognizes the physical environment [45] and are associated with a higher risk of accessibility problems in the home among older adults [14]. For older adults with difficulties recognizing the physical environment, it is considered important to have a familiar environment. Some types of housing adaptations that change the appearance of the environment may increase the risk of falling, because the individual may not be able to remember the familiar environment. Thus, older adults themselves and their LTC service professionals might not want to change the home environment [46]. Aspects such as dignity, autonomy, and independence are closely linked to the home [47], and decisions about housing adaptations are affected by many different considerations besides accessibility, such as the appearance of housing adaptations, the costs, and the space available in the home [48]. Such decisions should be respected and may, to some degree, explain the lower likelihood of housing adaptations related to visual and cognitive limitations.

However, these results could also imply that the existing types of housing adaptations in Japan do not meet the needs of older adults with lower cognitive functioning or visual impairments. For instance, older adults with cognitive impairment frequently need housing adaptations to lower the hot water temperature to avoid burns [49]. Similarly, older adults with visual impairments are advised to modify wall materials and lighting to ensure adequate lighting to support visibility [50, 51]. However, these types of housing adaptations are not readily available for older adults in Japan, because they are not included in the five types of housing adaptations that are granted. Thus, older adults cannot select these types of housing adaptations even if they have needs that would warrant them; therefore, policymakers need to pay attention to the housing environment not only for safety but also for accessibility by older adults with various functional limitations, especially those related to cognitive decline and visual impairment. This is in line with a recent Swedish study [52], which highlighted the need to amend current legislation to better support housing adaptations targeting cognitive limitations. The Swedish study also emphasized the need to develop new technology and design approaches that make the home environment more accessible for the growing number of older people who remain living in their own homes despite cognitive decline.

Our aim in this study is to examine whether housing adaptation grants are appropriately used by older adults with different types and levels of need; we found that older adults with public assistance were less likely to implement housing adaptation grants compared to older adults with an average level of household income. People receiving public assistance can apply for housing adaptations as long as they are certified by LTC insurance. Moreover, the 10% out-of-pocket payments for housing adaptations are paid as housing assistance for people who receive public assistance [53]; therefore, the burden of co-payments does not explain the low amount of housing adaptations. One possible reason is that 88.1% of people receiving public assistance live in rented housing [54] compared to only 22.9% of people who do not receive public assistance. This result can be partly explained by the current situation in which the resident requires approval from the owners to implement housing adaptations in rented housing. Moreover, this might be difficult because housing adaptation grants do not cover the costs to restore housing adaptations to the pre-adaptation design [15]. However, there is a need for further research to examine other reasons why people who receive public assistance do not obtain the housing adaptation grants to which they are entitled.

### Strengths and limitations of the study

A notable strength of this study is that we had access to comprehensive data covering all individuals certified for care support levels in one medium-large city in Japan, which supports the validity of inferences drawn from the results.

Our findings should be interpreted with three limitations in consideration. First, we cannot determine if some people did not require housing adaptations because their family had already implemented them owing to their functional decline, or they might have conducted housing adaptations before 2006. However, we used care needs certification for LTC insurance data from 2007 to 2011 to increase the appropriateness of the study sample and thus limit the inclusion of such individuals. Second, this study only uses LTC data; therefore, we cannot adjust for associated medical conditions. While we used specific medical procedures to address this, we could not adjust for the severity of underlying diseases that do not receive medical procedures. Third, individuals admitted to LTC facilities or residences for older adults with LTC services were included; therefore, residual confounding may occur in these cases. These facilities and residences have building standards that eliminate building hazards and ensure safety [55, 56], and therefore residents do not need to implement housing adaptation grants. However, the number of older adults staying at LTC facilities or residences is likely to be extremely limited because the applicants must be certified as LTC needs levels, which is more severe than that of our study participants [57, 58]. Moreover, people living in residences for older adults with LTC services are estimated to be only 4.3% of those who are certified as care support levels; therefore, the number of older adults with care support levels who admitted to these LTC facilities or residences would be very limited. Notwithstanding these limitations, this is the first study to consider housing adaptation services delivered from LTC insurance by focusing on functional limitations related to accessibility. In providing housing adaptation grants at the national level, policymakers need to reconsider the heterogeneity of older adults with functional limitations and the necessity to identify the types of adaptations that maximize their abilities.

## Conclusions

The results showed that older adults with cognitive or visual limitations implemented housing adaptations to a lesser extent, despite the high risk for accessibility problems in this population. Further research is warranted, as these results might suggest that housing adaptations are not used appropriately for all older adults with disabilities. Although the current study was conducted in a Japanese context, visual and cognitive limitations are strongly associated with age. With populations aging worldwide, the concern that these issues are not appropriately addressed may be similar in many countries. Housing adaptation grants should include the types of services that meet the needs of older people with all kinds of disabilities. The results may therefore indicate a need to improve the system that other countries should also look into.

## Supplementary Information

**Additional file 1: Supplementary Information 1.** The application of Japanese long-term care certification items to functional limitations related to housing accessibility. **Supplementary Information 2. **Timing of the implementation of housing adaptations during one year from care needs certification. **Supplementary Information 3.** The distribution of cost for housing adaptation during one year from care needs certification.

## Data Availability

All relevant data underlying this study are owned by the municipality’s project. There is an agreement between the municipality and the Dia Foundation for research on the ageing society. The agreement stipulates the following; this municipality does not allow the authors to use the data for any purpose other than this project or provide them to anyone other than the study members without permission from this municipality. Researchers interested in the data used here should contact the corresponding author, Dr. Tsuchiya-Ito.

## Abbreviations

AOR: Adjusted odds ratio
CI: Confidence interval
LTC: Long-term care
OR: Odds ratio
