# Supplementary material for: Implementation and costs of housing adaptations among older adults with different functional limitations in Japan
Source: BMC Geriatr. 2022 May 20;22:444. doi: 10.1186/s12877-022-03100-9 (PMC9123739; doi:10.1186/s12877-022-03100-9)
Supplement: Supplementary file 1 — Additional file 1: Supplementary Information 1. The application of Japanese long-term care certification items to functional limitations related to housing accessibility. Supplementary Information 2. Timing of the implementation of housing adaptations during one year from care needs certification. Supplementary Information 3. The distribution of cost for housing adaptation during one year from care needs certification. [file 12877_2022_3100_MOESM1_ESM.docx]

**Supplementary Information**

Supplementary Information 1. The application of Japanese long-term care certification items to functional limitations related to housing accessibility.

| Functional limitations related to accessibility ^a, b^ | | Items of Japanese long-term care certification |
| --- | --- | --- |
| Cognitive function | | |
|  | A. Difficulty in interpreting information ^a^  Dementia ^b^ | Degree of independent daily living for older people with dementia ^c^   - Independent - Rank I: Has some type of dementia, but almost independent in terms of daily living at home and in society. - Rank II: Some daily life-disturbing symptoms, behaviors and problems in communication seen but can lead daily life independently if kept watched by someone. IIa Condition II, mentioned above, seen outside home. IIb Condition II, mentioned above, seen at home. - Rank III: Daily life-disturbing symptoms, behaviors, and problems in communication that require assistance. IIIa Condition III, mentioned above, seen primarily during the daytime. IIIb Condition III, mentioned above, seen primarily at night. - Rank IV: Daily life-disturbing symptoms, behaviors, and problems in communication frequently require assistance. - Rank M: Marked psychiatric symptoms/related symptoms or serious physical disorders require expert management. |
| Visual impairment | | |
|  | B1. Visual impairment ^a^  B2. Blindness ^a^  Visual impairment ^b^ | Visual impairment  1. Intact (no disability in daily life)  2. Can see figure to check visual acuity from 1 m distance  3. Can see figure to check visual acuity in front  4. Hardly see anything  5. Cannot assess ^d^ |
| Hearing impairment | | |
|  | C. Loss of hearing ^a^ | Hearing impairment  1. Intact  2. Can hear normal volume of sound somehow  3. Can hear only loud sound  4. Hardly hear anything  5. Cannot assess ^d^ |
| Poor balance | | |
|  | D. Poor Balance ^a^ | One leg standing  1. Can keep standing without any support  2. Can keep standing with some support  3. Cannot keep standing |
| Upper extremity impairment | | |
|  | H. Reduced upper extremity function ^a^  J. Loss of upper extremity function ^a^ | Paralysis of upper extremity [right side/left side]  1. Existing paralysis  0. Not existing paralysis |
| Lower extremity impairment | | |
|  | K. Reduced spine and/or lower extremity function ^a^ | Paralysis of lower extremity [right side/left side]  1. Existing paralysis  0. Not existing paralysis |

*Footnotes*

- This application process was created by the authors.
- ^a^ Iwarsson, S., & Slaug, B. (2010). HOUSING ENABLER. Veten & Skapen & Slaug Enabling Development.
- ^b^ MacLachlan, M., Young Cho, H., Clarke, M., Mannan, H., Kayabu, B., & Ludolph, R. (2018). Web Annex F. Report of a systematic review on potential benefits of accessible home environments for people with functional impairments In: WHO housing and health guideline.
- ^c^ Kawagoe, S., Tsuda, T., & Doi, H. (2013). Study on the factors determining home death of patients during home care: A historical cohort study at a home care support clinic. Geriatrics & Gerontology International, 13(4), 874–880.
- ^d^ There were no data answering “cannot assess” in visual impairment and hearing impairment.

Supplementary Information 2. Timing of the implementation of housing adaptations during one year from care needs certification

*Footnotes*

- ^a^ This illustrated months between certified as care support levels and finalizing first housing adaptation. One month after long-term care certification was illustrated as “Month 1”.

Supplementary Information 3. The distribution of cost for housing adaptation during one year from care needs certification.

*Footnotes*

- The costs were illustrated in both JPY and USD using the exchange rate on March 31, 2018 (US$1 = \106.19).
- The maximum grants for housing adaptation in long-term care insurance are 200,000 JPY (1,883 USD), and the beneficiaries need to pay 10% of the housing adaptation costs; therefore, the maximum costs for housing adaptation in the claims were 180,000 JPY (1,695USD). Only those who have deteriorated in certified levels more than three levels or have moved can undergo housing adaptation again up to 200,000 JPY even if they have used the maximum amount of housing adaptation grants once.
